# A targeted circulating tumor DNA landscape of copy number aberrations in large B-cell lymphomas

**DOI:** 10.1038/s41375-026-02955-w

**Published:** 2026-04-22

**Authors:** Maare Arffman, Leo Meriranta, Judit Jørgensen, Marja-Liisa Karjalainen-Lindsberg, Klaus Beiske, Mette Pedersen, Kristina Drott, Øystein Fluge, Sirkku Jyrkkiö, Peter Brown, Harald Holte, Sirpa Leppä

**Affiliations:** 1 Research Programs Unit, Applied Tumor Genomics, University of Helsinki, Helsinki, Finland; 2 Department of Oncology, Helsinki University Hospital Comprehensive Cancer Centre, Helsinki, Finland; 3 Department of Hematology, Aarhus University Hospital, Aarhus, Denmark; 4 Department of Pathology, Helsinki University Hospital, Helsinki, Finland; 5 Department of Pathology, Oslo University Hospital, Oslo, Norway; 6 Institute of Clinical Medicine, Medical Faculty, University of Oslo, Oslo, Norway; 7 Department of Pathology, Zealand University Hospital, Roskilde, Denmark; 8 Department of Clinical Medicine, Faculty of Health and Medical Sciences, University of Copenhagen, Copenhagen, Denmark; 9 Department of Oncology, Skane University Hospital, Lund, Sweden; 10 Department of Oncology, Haukeland University Hospital, Bergen, Norway; 11 Department of Oncology, Turku University Hospital, Turku, Finland; 12 Department of Hematology, Rigshospitalet, Copenhagen, Denmark; 13 Department of Oncology, Oslo University Hospital, Oslo, Norway; 14 KG Jebsen Centre for B-cell malignancies, Oslo, Norway

**Keywords:** Translational research, B-cell lymphoma, Cancer genomics

## Abstract

The utility of circulating tumor DNA (ctDNA) for mutational genotyping, pretreatment prognostication, and assessment of molecular response is well established in patients with aggressive large B-cell lymphoma (LBCL). Here, we have applied targeted panel and duplex sequencing of plasma ctDNA to study copy number aberrations (CNAs) along with mutational landscapes in 123 uniformly treated patients with high-risk LBCL. We find a robust correlation between targeted and whole-genome sequenced CNA landscapes (*R* = 0.81) and identify CNAs in the ctDNA in 76% of the patients above the limit of detection. We describe the most frequently affected genomic regions, their interactions with diagnostic and genetic subtypes, and associations with overall and progression-free survival. Specifically, we show how ctDNA profiling of *TP53* loss outperforms fluorescence in situ hybridization (FISH)-based *TP53*/17p analysis in risk assessment, independent of clinical risk factors and ctDNA concentration. We validate key findings of prognostic tumor fraction and *TP53* loss in an independent LBCL cohort. Furthermore, we detect dynamic shifts between the fractions of lymphoma clones by assessing CNAs and mutations in the ctDNA at disease progression. These findings demonstrate the potential of minimally invasive, targeted CNA analysis in resolving the molecular heterogeneity of LBCLs.

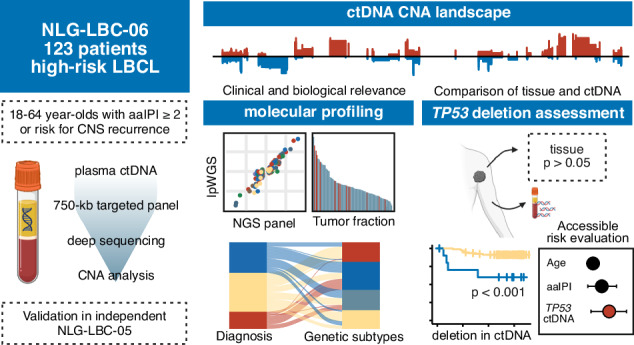

## Introduction

Large B-cell lymphomas (LBCLs) comprise a biologically and clinically heterogeneous group of the most common aggressive lymphoid cancers [1, 2]. Although patient outcomes are mainly favorable following anthracycline-based immunochemotherapy, 30% of patients will relapse after an initial response, with approximately 10% having primary refractory disease and being at high risk of dying from lymphoma [3]. On the other hand, many patients are overtreated and suffer from treatment-related toxicities [4]. Despite molecular characterization of LBCLs through the profiling of genetic landscapes [5–8], tumor microenvironment [9, 10], and host response [11], clinical and biological heterogeneity remains an obstacle to developing personalized treatments and improving survival.

The genomic landscape of LBCL is complex and comprises a diverse set of genetic alterations, including point mutations, translocations, and copy number aberrations (CNAs), which define genetic subtypes and impact patient outcomes [6–8, 12]. Among other aberrations, CNAs have an important role in the pathogenesis of LBCLs [13–15]. Specifically, the loss of *TP53* (17p13) is associated with increased resistance to chemotherapy and a poor prognosis [7, 8, 16]. In our trial, we also observed that, unlike *BCL2* and *MYC* translocations, the adverse impact of *TP53* deletion on survival could not be mitigated by dose-intensified treatment [17]. Other recurrent and potentially actionable aberrations, including loss of *CDKN2A* (9p21) or gain of *CD274* (9p24.1), have been associated with avoidance of immune response and more aggressive behavior in LBCLs [18, 19].

Recently, the detection of plasma circulating tumor DNA (ctDNA) has revolutionized LBCL profiling [20–22], and is expected to reduce the need for tissue biopsies. Targeted sequencing panels are often preferred in ctDNA profiling [20, 21, 23] to identify genetic abnormalities, primarily single nucleotide variants (SNVs) and small insertions and deletions. For instance, quantification of ctDNA burden at baseline [24–26] and the detection of minimal residual disease (MRD) [27–29], refined by high sequencing coverage together with duplex sequencing [17] or the identification of phased variants [23], have enabled dynamic risk assessment in LBCLs. However, profiling of CNAs from the ctDNA has remained less studied. A study employing shallow whole-genome sequencing showed that diffuse LBCL (DLBCL) not otherwise specified (NOS) can be distinguished from Hodgkin’s lymphoma using CNA profiles [30]. Another study using a targeted ctDNA panel showed that DLBCL NOS patients could be classified according to SNVs and CNAs into predefined genetic clusters, some of which, together with high ctDNA burden, were associated with worse survival [31]. In addition to the first-line therapy setting, the enrichment of specific CNAs has been investigated in CAR-T cell-resistant relapsed/refractory (R/R) LBCL patients [32]. While significant advances have been made in the field of lymphoma liquid biopsy (LB), the translational utility of ctDNA CNAs has not been addressed comprehensively in a uniformly treated cohort of LBCL patients.

The aim of this study was to identify clinically relevant CNAs in LBCL patients using a targeted panel designed for variant profiling and MRD testing, enabling multi-layer cfDNA profiling from the same LB source. To achieve this, we profiled plasma CNAs from 123 patients with primary LBCL treated in a Nordic phase II trial. We demonstrate the use of targeted ctDNA profiling for detecting CNAs, enhance risk assessment of LBCL patients, and validate key findings in another LBCL cohort. We describe subtype-specific molecular differences and report examples of the molecular background of expanding clones in patients with R/R DLBCL. Taken together, we provide clinically important CNAs that could be employed in the personalized treatment of LBCL patients.

## Materials and methods

### Patients and samples

#### Discovery cohort

The discovery cohort consisted of 123 patients aged 19-64 years with high-risk (advanced stage and age-adjusted International Prognostic Index (aaIPI) ≥ 2 and/or risk factors for central nervous system (CNS) recurrence) LBCL, who were treated in the Nordic Lymphoma Group (NLG)-LBC-06 trial (registered at ClinicalTrials.gov, trial number NCT03293173) [17]. The patients were treated with anthracycline-based intensified immunochemotherapy according to their biological risk factors (Figure S1A). The patient characteristics are described in Table 1.

**Table 1:** Patient characteristics.

Demographic	All patients (*n* = 123), n (%)	Patients with VAF^h^ ≥ 0.015 (*n* = 102), n (%)	Fisher's exact test, p-value
Age (years), median (range)	55 (19–64)	53 (20–64)	0.91 (Wilcoxon rank sum test)
Sex			1
Male	70 (57)	58 (57)	-
Female	53 (43)	44 (43)	-
Ann Arbor Stage			0.73
I-II	12 (10)	7 (7)	-
III	27 (22)	22 (22)	-
IV	84 (68)	73 (71)	-
B-symptoms	72 (59)	66 (65)	0.41
Elevated LDH^a^	107 (87)	92 (90)	0.53
aaIPI^b^			0.53
0-1	12 (9)	6 (6)	-
2	76 (62)	63 (62)	-
3	35 (29)	33 (32)	-
Bulky disease	46 (37)	40 (39)	0.9
Histology			1.00 (Histology)
DLBCL NOS^c^	102 (83)	83 (81)	0.88 (DLBCL NOS)
*GCB*^*d*^	47 (46)	36 (43)	-
*non-GCB*	54 (53)	46 (56)	-
*Unclassified*	1 (1)	1 (1)	-
HGBL^e^	14 (11)	12 (12)	-
THRLBCL^f^	4 (4)	4 (4)	-
FL^g^ grade 3B	3 (2)	3 (3)	-
Risk group			0.91
High risk	61 (50)	53 (52)	-
Low risk	62 (50)	49 (48)	-

^a^lactate dehydrogenase ^b^age-adjusted International Prognostic Index ^c^diffuse large B-cell lymphoma not otherwise specified ^d^germinal center B-cell ^e^high-grade B-cell lymphoma ^f^T-cell/histiocyte rich large B-cell lymphoma ^g^follicular lymphoma ^h^variant allele frequency.

For ctDNA analysis, plasma samples were available from 123, 116, 99, and 19 patients at baseline, after two or four cycles of therapy, at the end of treatment, and at follow-up, respectively. Sequential plasma samples were selected from four patients with R/R DLBCL. Matching pretreatment whole blood samples were available from 117 patients. Diagnostic formalin-fixed paraffin-embedded (FFPE) tumor tissues were available from 66 patients. The characteristics of the validation cohort are found in the Supplementary Materials and Methods.

All patients in the study signed informed consent before recruitment. The Institutional Review Boards, National Medical Agencies, and Ethics Committees in Finland, Norway, Denmark, and Sweden approved the protocols and sampling.

### ctDNA sequencing and copy number analysis

ctDNA was sequenced and variants were called from sequential plasma samples of 123 patients using a lymphoma-targeted 748-kb in-house NGS panel and duplex sequencing adapters (Table S1, Supplementary Materials and methods) [17, 33] at Finnish Institute of Molecular Medicine (FIMM; Helsinki, Finland). Sequencing data was segmented and CNAs called from on-target regions of ctDNA with Illumina’s Dragen Bio-IT copy number variant pipeline (version ≥ 4.2.4) at FIMM. Patients with a mean VAF ≥ 0.015 and autosomal CNAs with “Filter” = “PASS” were considered for further analysis (Table S2, Supplementary materials and methods).

### Statistical analyses

Analyses were performed in R (version ≥ 4.1.1) or conda (version > 24.11) environment. Statistical tests were, in general, non-parametric and two-sided. *P* values < 0.05 were considered statistically significant and marked in figures as follows: ns, *P* ≥ 0.05; *, *P* < 0.05; **, *P* < 0.01; ***, *P* < 0.001; ****, *P* < 0.0001.

## Results

### Patient characteristics

**Fig. 1:**
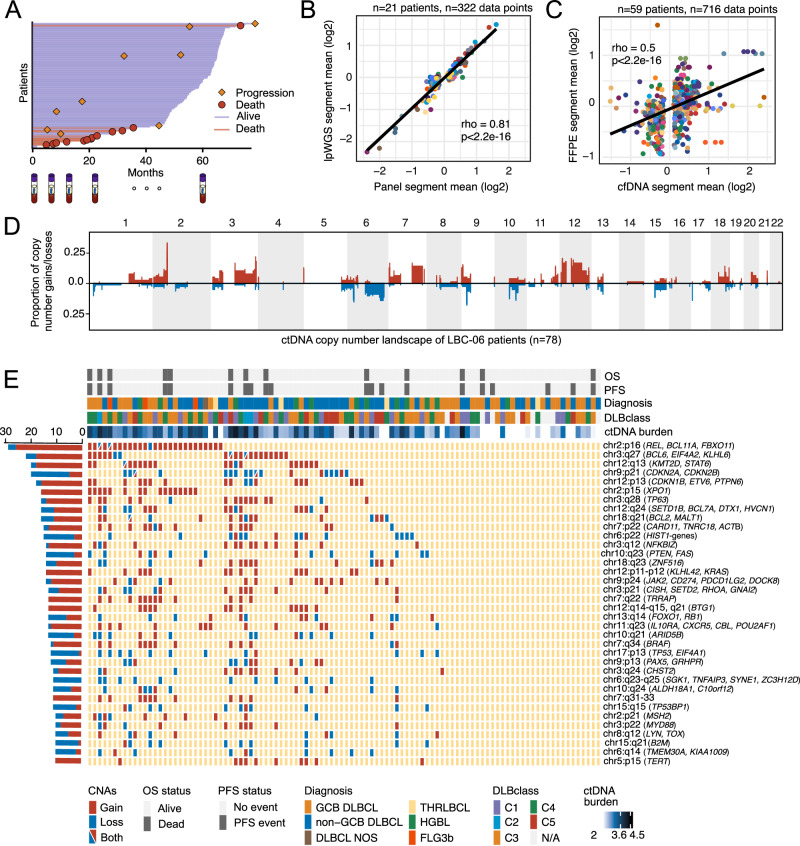
Targeted ctDNA CNA landscape of LBC-06 patients. **A** Swimmer plot of the patients in the LBC-06 trial (*n* = 123). Purple lines denote cured patients, whereas red lines denote patients who died. Disease progression and death events are depicted as orange squares and red spheres. **B** Spearman’s correlation of overlapping segment means (log2, *n* = 322) detected with targeted panel (x-axis) and low-pass WGS (y-axis). Patients (*n* = 21) are colored differently. **C** Spearman’s correlation of overlapping CNA segment means (log2) in the ctDNA  and segment means (log2) from matching FFPE tissues. Patients (*n* = 59) are colored differently. **D** Overall landscape of CNA proportions of *n* = 78 LBC-06 patients with *n* = 607 unique CNA segments across autosomal chromosomes. **E** Oncoprint of the most frequently aberrated regions. Gains and losses are colored with red and blue, respectively; copy number neutral regions are colored with yellow. Regions are annotated by cytobands, and the panel genes within these regions are marked in brackets. Every patient’s OS status, PFS status, diagnosis, DLBclass [39], and ctDNA burden are annotated on the oncoprint. lpWGS: low-pass whole genome sequencing, ctDNA: circulating tumor DNA, cfDNA: cell-free DNA, CNA: copy number aberration, OS: overall survival, PFS: progression free survival, GCB DLBCL: germinal center diffuse large B-cell lymphoma, NOS: not otherwise specified, THRLBCL: T-cell/histiocyte rich B-cell lymphoma, HGBL: high-grade B-cell lymphoma, FLG3b: follicular lymphoma grade 3B.

The patients (*n* = 123) included in the study were young (aged 18-64 years), had clinically high-risk (aaIPI ≥ 2 or site-specific risk factors for CNS recurrence) LBCL, and were treated uniformly in the Nordic phase II trial (Fig. 1A, Figure S1A, Table 1) with a dose-intensified immunochemotherapy [17]. The median follow-up time was 5 years at the time of the analysis. Plasma samples for ctDNA analyses were collected at baseline (BL), after two (CYC2) and/or after four cycles of therapy (CYC4), and at the end of treatment (EOT), and at follow-up (FU) (Fig. 1A, Figure S1A).

### Targeted sequencing enables the detection of CNAs in LBCL

We applied a duplex adapter-informed 748-kilobase targeted gene panel and Illumina’s Dragen calling pipeline to 123 pretherapeutic plasma samples (Table S1, Materials and Methods). We hypothesized that the targeted panel, designed primarily for mutational profiling and MRD assessment, could also detect biologically relevant and clinically significant CNAs. First, to test our hypothesis, we compared the CNAs from targeted sequencing with the copy number landscape obtained by low-pass whole-genome sequencing (lpWGS; *n* = 21 patients; Fig. 1B), a more traditional method for profiling CNAs in ctDNA [34–36]. We found that the raw log2 copy number values produced with targeted sequencing correlated robustly with those from lpWGS (rho = 0.81, Fig. 1B), providing good sensitivity and excellent specificity (0.709 and 0.964; Figure S1B). In detail, 5% (*n* = 133) of the segment calls were discordant between lpWGS and panel data (Figure S1C). Additionally, we found that the raw log2 copy number values correlated well with those obtained using another publicly available analysis pipeline [34] (rho = 0.72, Figure S1D). This enabled us to examine the CNAs from FFPE lymphoma tissues as well. The comparison of CNA landscapes between matching ctDNA and tumor tissue showed a moderate correlation (rho = 0.5, *n* = 59, Fig. 1C), likely to be weakened by not only the spatial restrictions of a tissue biopsy [20, 21], but also the noise in the FFPE data [37] (Figure S1E).

Next, as the ctDNA tumor fraction is reported to affect the CNA detection across cancers [38], we were interested in determining the limit of detection (LOD) of our approach. Indeed, we detected that ctDNA VAF was associated with the number of detected CNAs (Figure S1F-G). To mitigate the effect of low ctDNA content on copy number calling, we restricted the analyses to samples with a mean VAF ≥ 0.015, corresponding to a tumor fraction of 0.03 [34]. This resulted in the assessment of 102 patients. The patient demographics of the 102 patients were similar to those of the whole cohort (Table 1). Notably, the 21 patients with a mean VAF < 0.015 had low disease burden (Figure S1H), which was reflected in excellent survival (Figure S1I-J). Finally, a 1:2 in-silico down-sampling experiment of 11 plasma samples revealed that reducing sequencing depth decreased detectable CNAs in the ctDNA (Figure S1K-L, Supplementary Materials and methods), highlighting that, in addition to VAF, adequate sequencing coverage is important for detecting targeted CNAs in ctDNA.

Altogether, we sequenced plasma cfDNA from 123 LBCL patients and discovered that a targeted panel can be applied to analyze CNAs in ctDNA. Moreover, considering the effect of ctDNA content, we restricted the analyses to patients with a mean VAF ≥ 0.015, resulting in CNA assessment in 102 high-risk LBCL patients.

### CNAs in the ctDNA reveal biological heterogeneity in LBCLs

Next, we explored the CNA landscape (Fig. 1D, Table S2). Overall, the distribution of CNAs among patients was heterogeneous (Fig. 1D-1E), with the maximum number of individual CNAs captured by our targeted panel being 32 (mean 8). Out of 102 samples assessed, 78 (76%) had detectable CNAs, while 24 (24%) had no CNA calls (Fig. 1E, Table S2). The most recurrent CNAs in our data affected common aberrant genomic regions in DLBCL [6, 8, 12], including gains of 2p16, 3q27, and 12q13, which encompass the genes *REL*, *BCL6*, and *KMT2D*, respectively, and losses of 9p21 and 6p22, which affect *CDKN2A*, *CDKN2B*, and *HIST1* genes (Fig. 1E). When we combined CNAs with variant data, we found that specific mutations co-occurred with either copy number gains or losses (Figures S2A-B). For instance, losses co-occurred with coding mutations in genes such as *TP53*, *B2M*, and *HIST1H1E* (Figure S2B), suggesting a bi-allelic inactivation of these genes.

**Fig. 2:**
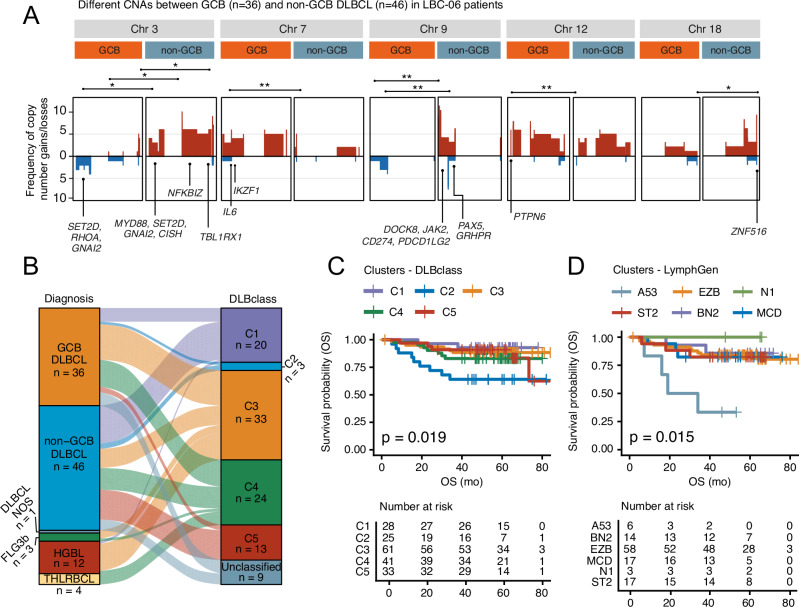
Subtype-specific heterogeneity detected by ctDNA CNAs. **A** Significantly different CNAs between GCB DLBCL (*n* = 36) and non-GCB DLBCLs (*n* = 46) in the discovery cohort. The frequency of CNAs per subtype is depicted in y-axis. P-value significance levels are marked on top, and genes are annotated at the bottom of the plot. **B** The diagnostic subtypes of the patients and their matched DLBclass subgroups [39]. DLBclass subgroups were analyzed from SNV, translocation, and CNA data. **C**, **D** Overall survival of study cohort and validation cohort together stratified by (**C**) DLBclass subtypes and (**D**) LymphGen subtypes. GCB DLBCL: germinal center diffuse large B-cell lymphoma, NOS: not otherwise specified, THRLBCL: T-cell/histiocyte rich B-cell lymphoma, HGBL: high-grade B-cell lymphoma, FLG3b: follicular lymphoma grade 3B; FISH: fluorescent in-situ hybridization, ctDNA: circulating tumor DNA, N/A: not available, OS: overall survival, mo: months.

To further examine molecular differences between LBCL patients, we investigated the CNAs by diagnostic subtypes. We detected subtype-specific CNAs between germinal center B-cell (GCB) and non-GCB DLBCL (Fig. 2A), prompting us to study whether these subtypes could be further characterized by minimally invasive genomic profiling. The assembly of somatic coding mutations, CNAs, and translocation profiles in ctDNA enabled us to implement the DLBclass [39] and LymphGen [6, 7] molecular clusters, both of which revealed genetic heterogeneity within the diagnostic LBCL subtypes (Fig. 2B, Figure S3A). Notably, we detected *BCL2* and *BCL6* translocations in ctDNA from multiple patients for whom FISH analysis was not available at diagnosis (Figure S3B). Apart from the discrepancy in the “Other” LymphGen subgroup caused by different confidence value thresholds, the two clustering methods mostly agreed on the genetic subtypes (Figure S3C), and the clustering confidence was comparable regardless of tumor fraction in the ctDNA (Figure S3D, cutoff appointed as per Chapuy et al. [39], Table S3).

To increase the statistical power of potentially prognostic molecular subtypes (Fig. 2B), we joined the molecular clustering data of the study cohort with data from an independent validation cohort (86 patients from the NLG-LBC-05 trial [40], who were treated with early high-dose methotrexate and dose-intensive immunochemotherapy, Supplementary Materials and Methods, Table S4). We found that the patients in the CNA-driven C2/A53 cluster had the worst OS and PFS (Fig. 2C-D, Figure S4A-B) and the highest tumor fraction in ctDNA (Figure S4C-D). Furthermore, the C2/A53 cluster remained an independent predictor of PFS after adjustment for ctDNA burden (Figure S4E), suggesting that although ctDNA abundance affects CNA calling, the clustering and prognostic effect are driven by the underlying molecular aberrations. When we conducted the DLBclass analysis only with coding mutations and structural variants, there was no difference in patient outcomes between the molecular subgroups (Figure S4F-G). These results, together with the requirement for CNA data for A53 inclusion in LymphGen classification, underscore the importance of CNA profiling for accurate subtype-specific survival prediction.

Taken together, we found CNAs in the ctDNA that uncover biological heterogeneity among established subtypes and enable detailed genetic subclassification of LBCL from LB. These results highlight the strong complementary potential of ctDNA for subtype characterization of LBCL patients when diagnostic tumor tissue is limited.

### High tumor fraction and multiple CNAs detect high-risk patients

**Fig. 3:**
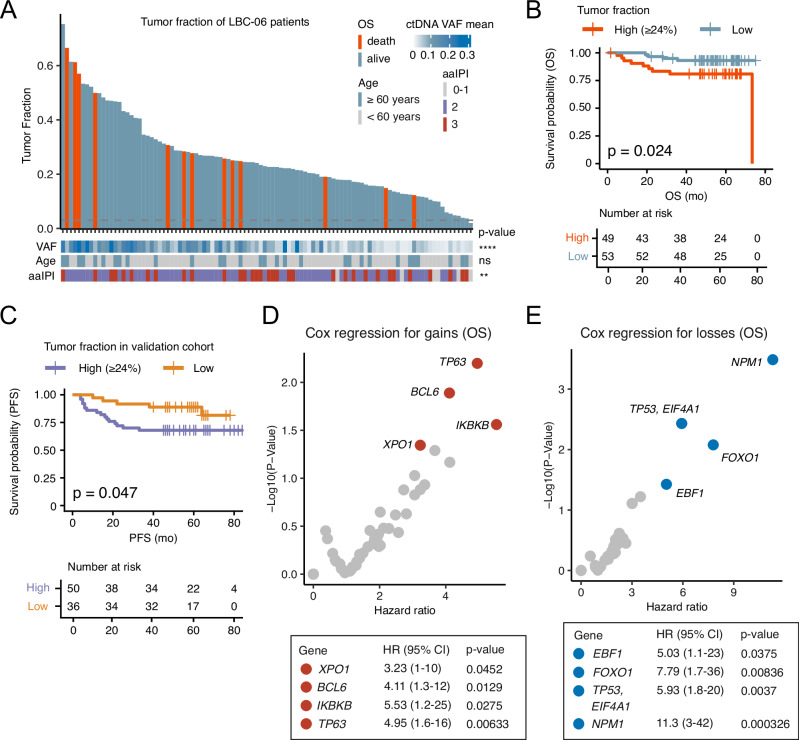
Characterization of high-risk LBCL patients using CNA landscapes. **A** Waterfall plot of tumor fractions in LBC-06 patients. Patients who died are colored orange, whereas patients who survived are colored blue. The gray dashed line represents a tumor fraction of 0.03, corresponding to the assay’s detection limit. Mean ctDNA VAF, age, and aaIPI are annotated at the bottom of the plot for each patient, and their correlation with tumor fraction is marked on the right. **B** Overall survival of patients stratified by tumor fraction. An optimal cutoff for tumor fraction (24%) was used as a threshold between patients with high and low tumor fractions. **C** Overall survival of validation cohort (LBC-05) stratified by tumor fraction. The discovery cohort’s cutoff for high tumor fraction (24%) was used to separate patients into high and low tumor fraction groups. **D**, **E** Univariate Cox regression analysis for OS of recurrent gains (**D**) and losses (**E**). The hazard ratio is depicted on the x-axis, and the -log10 p-value is on the y-axis. Aberrations in genes that reached statistical significance to OS are colored red (**D**) and blue (**E**). See also Table S5. ctDNA: circulating tumor DNA, VAF: variant allele frequency, OS: overall survival, PFS: progression-free survival, mo: months, aaIPI: age-adjusted International Prognostic Index, HR: hazard ratio, CI: confidence interval.

The survival association identified by genetic subgroups cued us to further explore associations between copy number landscapes and clinical characteristics. High copy number-derived tumor fraction in the LB has been shown to correlate with more aggressive DLBCL [41], and, likewise, we found that tumor fraction was associated with worse OS, high VAF in the ctDNA and aaIPI, but not with age (Fig. 3A, B) and was similar between panel-based and lpWGS data (*n* = 26, Figure S5A). We were able to validate the results in the validation cohort (Fig. 3C, Figures S5B-D). The estimates of tumor fractions were comparable between the discovery and validation cohorts (mean 0.26, range 0.02-0.75, and mean 0.30, range 0-0.86, respectively, Fig. 3A, Figure S5B), suggesting that high-risk LBCL patients have similar tumor fractions, enabling consistent risk assessment in the LB.

Next, we investigated whether individual CNAs could identify patients at high risk. By systematically applying the Cox regression model on recurrently altered regions ( ≥ 5 patients affected), we found that several CNAs, including *BCL6* gain and *TP53* loss, were associated with poor survival (Fig. 3D-E, Figures S5E-F, Table S5). The prognostic CNAs did not associate with clinical high-risk disease characteristics (Figure S5G), revealing heterogeneity within the clinical metrics used for patient stratification. Furthermore, we found that the number of prognostic CNAs cumulatively reflected survival (Figure S6A-B) and the C2 molecular subtype (Figure S6C). Harboring one or more of these high-risk CNAs remained as an independent prognostic factor for OS and PFS in multivariable analysis with age, aaIPI, and ctDNA concentration (Figure S6D-E).

Altogether, we observed similar tumor fraction landscapes in the LB and found that high tumor fraction is associated with worse survival in two independent patient cohorts. Additionally, we identified several prognostic CNAs that revealed clinical heterogeneity and found that harboring one or more of these aberrations was associated with worse survival cumulatively.

### TP53 loss reveals a clinically relevant group of patients

**Fig. 4:**
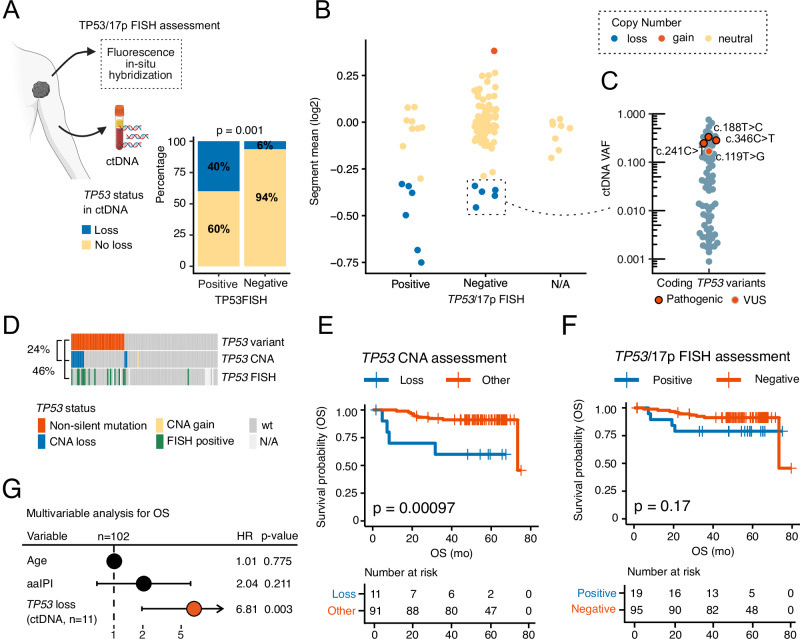
*TP53* status reveals a clinically relevant subgroup of LBCL patients. **A** Comparison of *TP53* status by two approaches: clinically used FISH from tumor tissue and ctDNA analysis. Chi-square test. Patient and plasma tube figures were created in BioRender.com, Arffman, M., 2025, https://BioRender.com/7xb3vjy. **B** Detailed differences in *TP53* statuses between tumor tissue FISH and ctDNA analyses. *TP53*/17p FISH status in x-axis and copy number segment mean (log2) in y-axis. Patients with *TP53* loss, *TP53* gain, and copy number neutral *TP53* in the CNA analysis are colored blue, orange, and yellow, respectively. **C** Non-silent *TP53* variants in the ctDNA and the variant allele frequencies in all patients. Orange points depict variants from patients with negative FISH *TP53*/17p status but loss in ctDNA with ClinVar estimates for variant pathogenicity. Blue points depict *TP53* variants from other patients. **D** Oncoprint of non-silent *TP53* variants in all patients together with *TP53* CNA and *TP53*/17p FISH statuses. Co-occurrence of the variant together with *TP53* loss is depicted on the left (only ctDNA-informed *TP53* loss: upper panel, both ctDNA- and FISH-informed *TP53* losses: lower panel). **E** Survival analysis for OS of the patients stratified by ctDNA *TP53* CNA status (*n* = 102). **F** Survival analysis for OS of all patients stratified by FISH *TP53*/17p status (*n* = 114). **G** Multivariable analysis for OS (*n* = 102): age, aaIPI, and ctDNA *TP53* loss (*n* = 11). FISH: fluorescent in-situ hybridization, ctDNA: circulating tumor DNA, N/A: not available, VAF: variant allele frequency, CNA: copy number aberration, IHC: immunohistochemistry, OS: overall survival, aaIPI: age-adjusted International Prognostic Index, HR: hazard ratio, PFS: progression-free survival, mo: months, VUS: variant of uncertain significance.

Our data had so far highlighted the loss of *TP53*/17p in both subtype clustering and as a prognostic factor. To further explore the translational potential of *TP53* loss, we examined it in the context of an already established marker used in risk stratification: a FISH-informed *TP53* status. Overall, the proportions of *TP53* statuses differed between the two assessment methods (chi-square test, p = 0.001, Fig. 4A). Upon closer examination, we found that several *TP53*/17p FISH-negative patients, indicating copy number neutrality, exhibited detectable *TP53* loss in their ctDNA (Fig. 4B). The same result was obtained with another CNA segmentation pipeline (Figure S7A-S7B). When we investigated patients with negative FISH results but CNA loss in their ctDNA, we found that all but one harbored a coding SNV in *TP53* (Fig. 4C). Overall, *TP53* mutations in ctDNA were mostly pathogenic [42] (Figure S7C), and 46% of patients with a *TP53* mutation had a *TP53* loss (Fig. 4D), suggesting a common LOH event in lymphomagenesis. Furthermore, the ctDNA *TP53* loss was associated with P53 positivity assessed by immunohistochemistry (Figure S7D). On the other hand, nine patients had *TP53*/17p FISH positivity despite undetected ctDNA loss (Fig. 4B). These patients had lower ctDNA concentration and tumor fraction than the patients with detectable *TP53* CNA (Figure S7E-S7F).

After detecting a discrepancy between ctDNA and FISH-informed methods, we next evaluated the prognostic value of these assessments. Even though we did not detect *TP53* losses in patients with small tumor fractions, ctDNA-assessed *TP53* loss was associated with poor survival (Fig. 4E, Figure S7G), and, notably, was more prognostic than FISH-informed *TP53*/17p status both for OS (Fig. 4E-F) and PFS (Figure S7G-S7H). ctDNA-informed *TP53* loss remained prognostic for OS in multivariable analysis with age and aaIPI (Fig. 4G, Table S6), whereas FISH-informed *TP53*/17p status did not (Figure S7I). Notably, ctDNA *TP53* loss remained an independent predictor for OS even after adjusting for ctDNA burden or tumor purity (Figure S7I, Table S6), underlining that despite the positive correlation with tumor content in cfDNA (Figure S7E-S7F), minimally invasive detection of *TP53* loss improved survival estimation. We observed that *TP53* loss in ctDNA was also associated with worse PFS in the validation cohort (Figure S7J). Finally, although the co-occurrence of coding *TP53* mutations and *TP53* loss in ctDNA was associated with poor survival (Figure S7K), it did not improve survival assessment compared to CNA-based assessment alone. The results suggest that *TP53* loss in ctDNA can serve as an independent marker to identify high-risk patients.

Altogether, these results demonstrate that LB-based *TP53* assessment improves risk stratification over traditional a tissue-based method, enabling more accessible yet clinically relevant molecular profiling.

### Copy number profiles and cancer cell fractions reveal structures of lymphoma progression

**Fig. 5:**
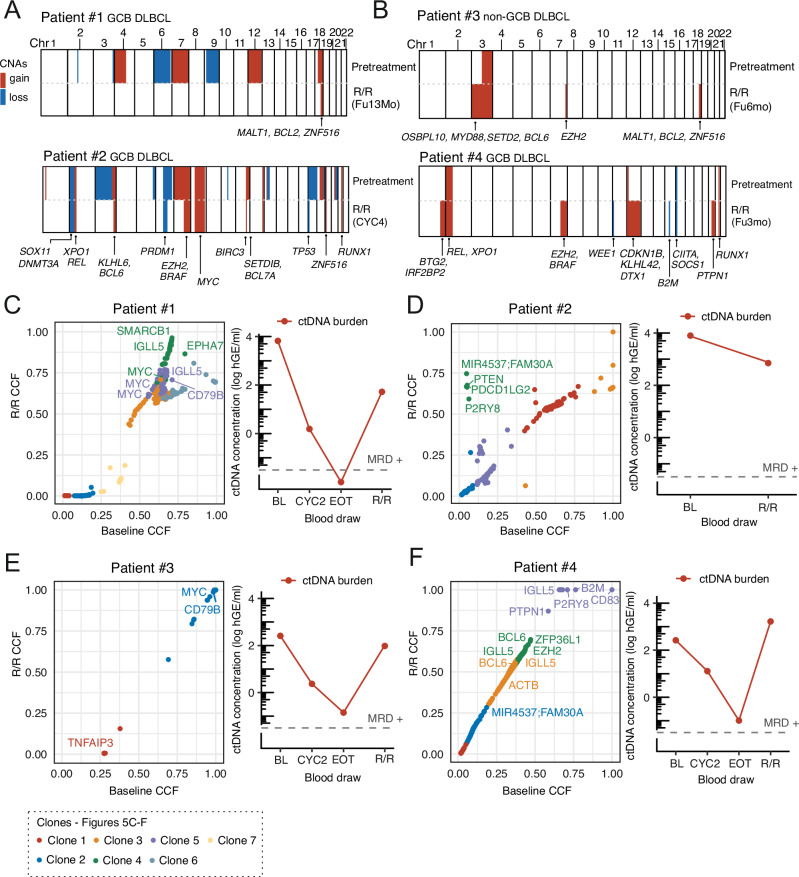
Analysis of clonal population structures in R/R patients. **A**, **B** CNA landscapes of patients with less CNAs (Patient #1 and Patient #2, **A**) and more CNAs (Patient #3 and Patient #4, **B**) in the R/R plasma sample compared to pretreatment plasma sample. Selected genes are annotated below the landscape plots. **C** Patient #1 molecular dynamics: cancer cell fractions of distinct clones before treatment and at disease progression (left panel). Clones are marked with different colors, and selected genes in significantly expanding clones are annotated. ctDNA concentration at baseline, throughout treatment, and at disease progression (right panel). The gray dashed line at -1.5 denotes the threshold for MRD test positivity. **D** Patient #2 molecular dynamics: cancer cell fractions of distinct clones before treatment and at disease progression (left panel). Clones are colored with different colors, and selected genes in significantly expanding clones are annotated. ctDNA concentration at baseline, throughout treatment, and at disease progression (right panel). The gray dashed line at -1.5 denotes the threshold for MRD test positivity. **E** Patient #3 molecular dynamics: cancer cell fractions of distinct clones before treatment and at disease progression (left panel). Clones are marked with different colors, and selected genes are annotated. ctDNA concentration at baseline, throughout treatment, and at disease progression (right panel). The gray dashed line at -1.5 denotes the threshold for MRD positivity. **F** Patient #4 molecular dynamics: cancer cell fractions of distinct clones before treatment and at disease progression (left panel). Clones are marked with different colors, and selected genes in significantly expanding clones are annotated. ctDNA concentration at baseline, throughout treatment, and at disease progression (right panel). The gray dashed line at -1.5 denotes the threshold for MRD test positivity. GCB DLBCL: germinal center diffuse large B-cell lymphoma, CNA: copy number aberration, CCF: cancer cell fraction, R/R: relapsed/refractory, BL: baseline, CYC2: after 2 cycles, EOT: end of treatment, MRD: minimal residual disease, FU: follow-up, Mo: month.

Finally, we were interested in studying ctDNA dynamics in sequential samples. Although most patients had no detectable CNAs in samples obtained during or after therapy due to diminished ctDNA levels, we wondered if the copy number landscape could inform about the mutational structure of non-responding lymphomas. Accordingly, while some patients with R/R LBCL showed a reduction in their CNAs during therapy (Patients #1-#2, Fig. 5A), others acquired new CNAs (Patients #3-#4, Fig. 5B). These results indicate that LBCLs adopt distinct mutational mechanisms in progression.

To dynamically model cancer cell population structures across different time points [43], we combined CNA and SNV data from the ctDNA. Strikingly, duplex correction enabled highly detailed characterization of the cancer cell fraction (CCF) of the distinct clones (Figure S8A-B). Despite MRD negativity at the EOT (Patient #1), the main clones at pre-treatment were also frequently prominent at progression (Fig. 5C-F). However, we found shifts in CCFs between the two time points in all patients (Figure S8C). For example, a significant expansion of clones that harbored mutations in *MYC* and *CD79B* (Patient #1, Fig. 5C), *PDCD1LG2* (Patient #2, Fig. 5D), and *B2M, CD83*, and *IGLL5* (Patient #4, Fig. 5F) was detected (Figure S8C). Furthermore, emerging CNAs in patients #3 and #4 (Fig. 5B) that did not overlap with any SNVs suggest that there are additional clonal characteristics beyond SNV-dependent CCF estimates.

Collectively, these findings indicate that lymphoma evolution is a dynamic process, which can be revealed by joint analysis of CNAs and smaller variants in the LB. Although descriptive and mostly hypothesis-generating, these examples highlight the expanding opportunities for minimally invasive profiling in a sequential setting and suggest that, despite frequent fluctuations in CCFs, disease progression may often be driven by the founding clone.

## Discussion

Risk stratification has remained a challenge in LBCL [3], and ctDNA profiling has become an increasingly common approach to address it. With a growing number of detection methods capable of detecting cancer-derived signals in blood samples, targeted sequencing panels are frequently used to reveal mutational landscapes [21], and are essential for measuring tumor burden and MRD kinetics in LBCL patients [23]. Although targeted panels include information on various genetic alterations, their use for CNA detection in ctDNA remains less established. Here, we profiled CNA landscapes using a targeted NGS panel, which revealed clinically and biologically relevant heterogeneity in a uniformly treated LBCL patient cohort. Further, we were able to validate key findings of prognostic tumor fraction and *TP53* loss in the ctDNA in an independent cohort and describe shifting clonal population structures at disease progression.

Although the prognostic role of ctDNA levels is well recognized and therefore used to risk-stratify patients according to tumor burden or MRD, there are no universal thresholds established for high-risk patients [44]. This is primarily due to heterogeneity in patient cohorts and the methodologies used for variant detection. Moreover, uncertainties in variant calling, such as those posed by polymorphisms or clonal hematopoiesis, might complicate ctDNA analysis. Our study demonstrated that, in addition to prognostic tumor fraction and survival-associated gene-level CNAs, the profiling of *TP53* status in ctDNA outperformed routine *TP53/17p* FISH assessment, while remaining independent of clinical risk factors, ctDNA burden, and tumor fraction. Notably, the FISH-informed *TP5*3/17p status was no longer prognostic at 5-year follow-up, updating our previous results [17]. Accordingly, *TP53* loss in the LB could be applied to complement the prognostic toolkit of ctDNA for high-risk lymphoma patients that could benefit from biologically targeted therapies, such as the addition of decitabine [45–47]. Low-purity cfDNA samples, however, may fail to meet the LOD required for CNA assessment, a challenge well recognized across algorithms and various cancers, including lymphoma [48, 49]. We likewise acknowledge that 21 patients were excluded from the study due to low ctDNA content, and further, that 24% of the 102 included patients did not have detectable CNAs in ctDNA. Therefore, it is likely that some clinically important CNAs are missed by our approach. Nevertheless, in our study, patients who did not meet the LOD or those with a low tumor fraction had excellent survival. Therefore, copy number profiling in the LB might be an attractive option for patient risk stratification.

Besides risk-adapted treatment, distinct molecular subtypes are used to stratify LBCL patients [6–8, 39]. A recent study demonstrated a survival benefit of subtype-targeted therapy compared to R-CHOP in DLBCL patients [46]. The trial, however, relied solely on tumor biopsies and did not include any CNAs in the simplified 20-gene algorithm. We show that combining CNAs with other variants in ctDNA enables the assignment of patients into subtype-specific clusters. Furthermore, our results showed, in line with previous findings [7, 12], that ignoring CNAs in the algorithm compromises the C2/A53 cluster during assessment. This affected the survival estimates, as the CNA-heavy patients were evenly spread between the remaining clusters. We recognize the differing subtype-specific survival estimates between our study and previous findings [7]. However, our prior observations [17, 40] of dose-intensive immunochemotherapy overcoming the adverse prognostic impact of MCD/N1 genomic subtypes, double-hit lymphomas as well as ABC phenotype remain consistent even with the more comprehensive and confident subtype classification, which benefits [39] from the addition of NGS-profiled CNAs. Conclusively, we highlight the importance of CNA analysis in minimally invasive subtype stratification.

We profiled ctDNA CNAs using 748-kb and 235-kb panels in discovery and validation cohorts. Although validating the findings in another LBCL cohort emphasizes the use of targeted CNAs as a minimally invasive prognostic marker, the use of a smaller panel with lower sequencing depth was reflected in the quality of CNAs in the validation cohort. While our discovery cohort’s panel performed optimally, the validation cohort’s panel tended to overestimate losses, hindering a comprehensive analysis of CNAs in the ctDNA. These results imply that a deeply sequenced 748 kb panel, which is optimal for MRD detection [17, 33], also provides a platform for copy number analysis. A recent study used a 559 kb panel to estimate ctDNA tumor fractions, similarly to our study, and found a high positive correlation with WGS profiling [41]. Therefore, we conclude that CNAs reveal clinically important heterogeneity and recommend profiling of CNAs in ctDNA with targeted panels.

Our study has several limitations. First, because we used a targeted panel, our approach did not account for the exact number of CNAs, and the sensitivity of the targeted assay was modest compared to that of the lpWGS data. Second, we analyzed only autosomal CNAs, excluding potential CNAs in the sex chromosomes. Lastly, our pipeline did not adjust CNA calling for tumor purity, which may have led to the underestimation of CNAs in samples with low ctDNA fractions, leaving, for example, our exploration of clonal evolution largely anecdotal. Nevertheless, our findings provide a clinically applicable tool for characterizing high-risk LBCL patients, which could be employed for other cancers with similar clinical challenges.

In summary, we present CNA landscapes in the ctDNA from 123 patients treated in a Nordic lymphoma trial. The strengths of our study are a homogenously treated high-risk LBCL patient cohort with systematically collected plasma samples and clinical data. Additionally, we validate key findings with an independent LBCL cohort with similar demographics, treatment, and follow-up data. We describe the landscape of the most frequently altered genomic regions and combine multiple ctDNA levels to cluster patients by biological subtypes. We reveal survival-associated CNAs that could complement patient risk stratification alongside other LB-based estimates. We predict increased use of CNA profiling from ctDNA for unveiling heterogeneity in lymphoma and hypothesize that targeted CNA landscapes can be used in clinical decision-making.

## Supplementary information

Supplementary material

Table S1.

Table S2.

Table S3.

Table S4.

Table S5.

Table S6

## Data Availability

In accordance with European Union legislation, the General Data Protection Regulation (GDPR) and the Finnish Act on the Secondary Use of Health and Social Data, the clinical annotations and the linked sequencing data from this study cannot be deposited or shared publicly due to the sensitive patient data protection. Collaboration agreements, compliant with the above and accepted by the local ethics committees and authorities, can be queried from the last author. Processed sequencing data supporting the findings of this study are available in the supplementary materials.

## Prior presentation

Part of the work has been presented at the AACR Annual Meeting 2025.
