# Supplementary material for: A targeted circulating tumor DNA landscape of copy number aberrations in large B-cell lymphomas
Source: Leukemia. 2026 Apr 22;40(7):1381–91. doi: 10.1038/s41375-026-02955-w (PMC13323072; doi:10.1038/s41375-026-02955-w)

Supplementary Material

A targeted circulating tumor DNA landscape of copy number aberrations in large B-cell lymphomas

Maare Arffman[1,2], Leo Meriranta[1,2], Judit Jørgensen[3], Marja-Liisa Karjalainen-Lindsberg[4], Klaus Beiske[5,6], Mette Pedersen[7,8], Kristina Drott[9], Øystein Fluge[10], Sirkku Jyrkkiö[11], Peter Brown[12], Harald Holte[13,14], Sirpa Leppä[1,2]

[1]Research Programs Unit, Applied Tumor Genomics, University of Helsinki, Helsinki, Finland
[2]Department of Oncology, Helsinki University Hospital Comprehensive Cancer Centre, Helsinki, Finland
[3]Department of Hematology, Aarhus University Hospital, Aarhus, Denmark
[4]Department of Pathology, Helsinki University Hospital, Helsinki, Finland
[5]Department of Pathology, Oslo University Hospital, Oslo, Norway
[6]Institute of Clinical Medicine, Medical Faculty, University of Oslo, Oslo, Norway
[7]Department of Pathology, Zealand University Hospital, Roskilde, Denmark
[8]Department of Clinical Medicine, Faculty of Health and Medical Sciences, University of Copenhagen
[9]Department of Oncology, Skane University Hospital, Lund, Sweden
[10]Department of Oncology, Haukeland University Hospital, Bergen, Norway
[11]Department of Oncology, Turku University Hospital, Turku, Finland
[12]Department of Hematology, Rigshospitalet, Copenhagen, Denmark
[13]Department of Oncology, Oslo University Hospital, Oslo, Norway
[14]KG Jebsen Centre for B-cell malignancies, Oslo, Norway

Running title: Cell-free copy number aberrations in B-cell lymphomas

Keywords: liquid biopsy, circulating tumor DNA, copy number aberrations, large B-cell lymphoma

List of Supplementary Tables

Table S1. Panel genes and coordinates. On-target regions of the panel used for sequencing. Reported and filtered genes (quality control, QC) for copy number data analysis.

Table S2. ctDNA CNAs in LBC-06 patients. All quality filtered (Filter == PASS) ctDNA CNAs in LBC-06 patients.

Table S3. Molecular subtypes of LBC-06 patients. DLBclass and LymphGen clusters for LBC-06 patients.

Table S4. ctDNA CNAs in LBC-05 patients. CNAs profiled with ichorCNA against process matched healthy donor plasma cfDNA samples.

Table S5. Overall survival associating CNAs. Losses are colored blue and gains are colored red.

Table S6. Multivariable analysis for overall survival.

Supplementary materials and methods

Patients

Discovery cohort

The discovery cohort of 123 large B-cell lymphoma patients was treated in a phase II Nordic trial[1] with risk-adapted anthracycline-based immunochemotherapy. All patients received two courses of R-CHOP-21 with interpolated HD-Mtx. Depending on the biological risk factors, the treatment was continued with either four courses of R-CHOEP-14 (no risk factors; R-CHOP-14 and etoposide), or four dose adjusted (DA)-EPOCH-R courses. Additionally, all patients received one course of R-HD-cytarabine. Biological high-risk factors included the presence of *C-MYC* translocation, *C-MYC* and *BCL2* translocation (double hit), *17p/TP53* deletion, co-expression of MYC and BCL2, P53 positivity, and/or CD5 positivity. Patient characteristics are presented in Table 1.

Validation cohort

The validation cohort consisted of high-risk LBCL patients treated in the NLG-LBC-05 phase II trial (registered at ClinicalTrials.gov with trial number NCT03293173)[2]. The cohort had similar inclusion criteria to those of the discovery cohort, except that primary mediastinal B-cell lymphomas (n=8) were included. Additionally, there was no prospective stratification according to biological risk factors, and all patients received the same systemic immunochemotherapy as the biological low-risk group in the discovery cohort. Pretreatment plasma samples[3] for cfDNA assessment were available from 100 patients.

ctDNA analysis

Sequencing and variant calling

Circulating tumor DNA (ctDNA) sequencing and variant calling from sequential plasma samples was profiled from 123 patients using a lymphoma driver, immunoglobulin and clonal hematopoiesis of intermediate potential (CHIP) region targeting 748 kilobase (kb) in-house NGS-panel and duplex sequencing adapters[1, 4]. Briefly, ten milliliters of peripheral blood were drawn into standard K2- or Streck-EDTA tubes, centrifuged, aliquoted, and stored until cell-free DNA isolation. Cell-free DNA was extracted from plasma samples using QIAseq cfDNA Extraction Kit (Qiagen, Hilden, Germany) according to protocol instructions. Whole blood was drawn in K2-EDTA tubes, aliquoted, and stored until isolation with QIAamp DNA Blood Midi Kit (Qiagen, Hilden, Germany). Up to 50 nanograms of formalin-fixed paraffin-embedded (FFPE) or fresh frozen (FF) tumor tissue were extracted with QIAGEN's QIAamp DNA FFPE Advanced UNG Kit and QIAamp Fast DNA Tissue Kit, respectively. Sequencing libraries were constructed for cfDNA using IDT xGEN Duplex Seq adapters (Integrated DNA Technologies, Coralville, Iowa, US). Biotinylated probes were used for target enrichment covering common lymphoma and CHIP drivers, regions of somatic hypermutation and immunoglobulin genes. Sequencing was done with the Novaseq6000 (Illumina, San Diego, CA, USA) instrument with hg38 reference in mapping. Polymorphisms were suppressed against the matched normal sample, and diagnostic tumor tissue was used for variant calling, together with pretreatment ctDNA when available. The pathogenicity of coding *TP53* variants was assessed with ClinVar classifications[5]. Variants with pathogenic, likely pathogenic, strong or potential somatic evidence (type I and II) and oncogenic or likely oncogenic somatic variants were reported as “pathogenic”, whereas others were deemed as “not pathogenic”.

Translocations (SVTYPE = BND) were called from the ctDNA using Illumina’s Dragen Bio-IT (version ≥ 4.2.4) pipeline at Finnish Institute of Molecular Medicine (FIMM; Helsinki, Finland). Quality filtered (Filter = ”PASS”) regions of *BCL2* (18q21.33) and *BCL6* (3q27.3) with ≥ 3 alternative split or paired reads and any evidence of reference reads were considered for the analysis after case by case confirmation on Integrative Genomics Viewer (version 2.18.4).

cfDNA copy number profiling

ctDNA copy number analysis

ctDNA copy number analysis was performed from on-target reads determined by our 748-kb target panel (Table S1) with Illumina’s Dragen Bio-IT (version ≥ 4.2.4) somatic copy number pipeline at FIMM. Target counts were produced using signals on read depth and improper pairs, after which the samples were normalized, segmented, and copy number aberrations (CNAs) were called. In normalization, samples were GC-corrected and normalized against a panel of normal (PoN) compiled from 17 individual plasma samples from healthy volunteers. Copy number segments that overlapped with recurrently aberrated regions in another set of healthy controls sequenced in parallel (n=10) were filtered out as polymorphisms. To alleviate the effect of cfDNA concentration to CNA calling, a previously suggested threshold of variant allele frequency (VAF) ≥ 0.015 (0.03 tumor fraction)[6] was used. After VAF filtering, autosomal CNAs with “Filter” = “PASS” were considered for further analysis (Table S2).

Low-pass whole genome sequencing (WGS, mean 2.9x) was performed to the pretreatment samples of 26 patients. Patients were selected to represent different tumor burdens, age,

diagnostic subtypes and clinical risks. From these, 21 samples had detectable CNAs, which were produced against the sequencing unit's in-house PoN, analyzed with Dragen and correlated to CNAs generated with the targeted panel. Five samples did not have any detectable CNAs by lpWGS. Tumor fraction estimates were performed with ichorCNA (version 0.3.2) for all lpWGS samples using the algorithm's built-in PoN [6].

In-silico down sampling series

The in-silico dilution series was performed for 11 baseline samples by down-sampling sequencing files to 0.5, 0.25, 0.125, 0.0625 and 0.03125 proportion of the original file. Segments that overlapped at least 95% between the original and the diluted sample were considered as matching segments.

Validation of ctDNA CNA landscapes

Validation of ctDNA CNAs was performed with ichorCNA[6] (version 0.3.2). All samples were GC-corrected and analyzed against a reference mappability score track. The samples of the study and validation cohorts were run against hg38 and hg19 track files, respectively. Segmentation was performed in 500kb bins. For technical validation of the discovery cohort, all 123 baseline ctDNA samples were analyzed. A PoN of 10 individual plasma samples from healthy volunteers was used for normalization. Tumor tissue CNAs from the discovery cohort were analyzed from FFPE (n=66) samples. Process- and patient-matched whole blood samples (n=65) were used as an input for a PoN. For the validation cohort (Table S4), reads overlapping with genes *KLHL6* and *DTX1* were removed due to traces of contamination. 100 ctDNA samples were analyzed with a 235-kb gene panel, but to reflect the discovery cohort better, further analyses were restricted to

patients with a mean VAF ≥ 0.015 (n=86). Eight individual samples from healthy volunteers were used to construct the PoN for the validation cohort.

Survival analysis

All survival analyses between patient groups were done with Kaplan-Meier log rank test using R package *survival* (version 3.8.3) and visualized with *survminer* (version 0.5.1). Cox regression was used to estimate survival in both univariable and multivariable models, utilizing the R package *survival*. Tumor fraction high and low groups were determined with the *maxstat* package (R, version 0.7.26) by finding the maximal cut point to predict OS for the discovery cohort (optimal cutoff 0.24). Landscape plots of CNAs were generated with *GenVisR* (R, version 1.39.0) and *copynumber* (R, version 1.29.0.9000), heatmaps with *ComplexHeatmap* (R, version 2.24.1) and co-occurrence plots with *UpSetR* (R, 1.4.0).

Molecular subtype classification

DLBclass classification

Patients were assigned to mutational DLBclass subgroups with DLBClass tool (version 1.0.0) according to the instructions by Chapuy et al[7]. A gene sample matrix (GSM) was constructed using somatic coding mutations and CNAs detected from the ctDNA, and structural variants from FISH analysis. Structural variants for BCL2 and BCL6 were complemented with data from ctDNA. After matching available data with the GSM input requirements, DLBclass classification was performed with 122 features. For somatic mutations, the value “1” was used for silent mutations and the value “2” was used for non-silent mutations. Segments with CNAs from the ctDNA targeted panel sequencing were annotated with cytoband nomenclature. High-level copy number

gains (Segment Mean > 1.9) were assigned with value “2”, whereas value “1” was used for other, low-level copy number gains. All losses were low-level copy number losses (0.1 < CR < 0.8) and therefore assigned with “1”. Arm-level copy number loss of 17p was performed with FISH analysis and assigned with “1”. No other arm-level CNAs were assessed. Value “3” was used for detected structural variants (SV.MYC, SV.BCL2, SV.BCL6). Value “0” was used to indicate the absence of any mutation. In total, 106 patients were classified to C1-C5 subgroups; 17 patients had five of fewer GSM features and were set as “unclassified”. Predictions with value ≥0.70 were assigned as high confidence.

LymphGen classification

LymphGen subgroups[8, 9] (version 2.0) were predicted according to the developers’ instructions from all coding and 5’UTR variants in the ctDNA. Structural variant data of *BCL2* and *BCL6* were compiled both from ctDNA and tumor tissue FISH analysis, similarly to the DLBclass assessment. For CNAs, ctDNA data was used with “HETLOSS and GAIN only” option. All subgroups were assessed. Predictions with value ≥0.70 were assigned as high confidence.

For the validation cohort, DLBclass and Lymphgen subgroups were assigned with following differences: translocations were only available from tumor tissue, and no arm-level copy number losses for 17p were available. Other features were composed similarly as in the study cohort.

Assessment of clonal structures

Assessment of clonal structures from variant and copy number data was done with PyClone[10] using Python version 2.7.18. Force called single nucleotide variants (SNVs) that were profiled in all sequential samples were used as input. All SNVs in CHIP genes, including *ASXL1*, *CBL*, *DNMT3A*, *FLT3*, *GNAS*, *IDH1*, *IDH2*, *JAK2*, *NPM1*, *PPM1D*, *RUNX1*, *TET2*, and *TNRC18* were filtered out from the analysis. Absolute copy number was determined by the presence or absence of overlapping CNAs and set to major_cn. As we did not have information on allele-specific CNAs, prior was set to total copy number (-prior total_copy_number). Burnin was set to 1000 (-burnin 1000), number of iterations to 10000 (-num_iters 10000), and default settings were used for all other parameters. Clusters were tidied as follows: they were pooled according to the closest mean cellular prevalence until a minimum cluster size of 3 was reached, as previously shown[11]. Moreover, to determine cancer cell fractions, the cellular prevalence of each variant was divided by the cellular prevalence of the sample's cluster with the highest mean cellular prevalence. After this, the CCF of variants with a CCF >1 was capped to 1.

References

1. Leppa S, Meriranta L, Arffman M, Jorgensen J, Karjalainen-Lindsberg ML, Beiske K, et al. Biomarker-adapted treatment in high-risk large B-cell lymphoma. Hemasphere. 2025;9(5):e70139.
2. Leppa S, Jorgensen J, Tierens A, Meriranta L, Ostlie I, de Nully Brown P, et al. Patients with high-risk DLBCL benefit from dose-dense immunochemotherapy combined with early systemic CNS prophylaxis. Blood Adv. 2020;4(9):1906-15.
3. Meriranta L, Alkodsi A, Pasanen A, Lepisto M, Mapar P, Blaker YN, et al. Molecular features encoded in the ctDNA reveal heterogeneity and predict outcome in high-risk aggressive B-cell lymphoma. Blood. 2022;139(12):1863-77.
4. Meriranta L, Rask Kragh Jorgensen R, Pasanen A, Kolstad A, Hutchings M, Niemann CU, et al. Circulating Tumor DNA Determinants of Response and Outcome in Relapsed/Refractory Mantle Cell Lymphoma. Blood Adv. 2025.
5. Landrum MJ, Lee JM, Riley GR, Jang W, Rubinstein WS, Church DM, et al. ClinVar: public archive of relationships among sequence variation and human phenotype. Nucleic Acids Res. 2014;42(Database issue):D980-5.
6. Adalsteinsson VA, Ha G, Freeman SS, Choudhury AD, Stover DG, Parsons HA, et al. Scalable whole-exome sequencing of cell-free DNA reveals high concordance with metastatic tumors. Nat Commun. 2017;8(1):1324.
7. Chapuy B, Stewart C, Dunford AJ, Kim J, Kamburov A, Redd RA, et al. Molecular subtypes of diffuse large B cell lymphoma are associated with distinct pathogenic mechanisms and outcomes. Nat Med. 2018;24(5):679-90.
8. Schmitz R, Wright GW, Huang DW, Johnson CA, Phelan JD, Wang JQ, et al. Genetics and Pathogenesis of Diffuse Large B-Cell Lymphoma. N Engl J Med. 2018;378(15):1396-407.
9. Wright GW, Huang DW, Phelan JD, Coulibaly ZA, Roulland S, Young RM, et al. A Probabilistic Classification Tool for Genetic Subtypes of Diffuse Large B Cell Lymphoma with Therapeutic Implications. Cancer Cell. 2020;37(4):551-68 e14.
10. Roth A, Khattra J, Yap D, Wan A, Laks E, Biele J, et al. PyClone: statistical inference of clonal population structure in cancer. Nat Methods. 2014;11(4):396-8.
11. Sobesky S, Mammadova L, Cirillo M, Drees EEE, Mattlener J, Dorr H, et al. In-depth cell-free DNA sequencing reveals genomic landscape of Hodgkin's lymphoma and facilitates ultrasensitive residual disease detection. Med. 2021;2(10):1171-93 e11.
12. Chapuy B, Wood T, Stewart C, Dunford A, Wienand K, Khan SJ, et al. DLBclass: A Probabilistic Molecular Classifier to Guide Clinical Investigation and Practice in DLBCL. Blood. 2025;145(18):2041-55.

Supplementary Figure legends

Figure S1. Assessment of CNAs with targeted panel

A) Schematic overview of the LBC-06 trial.

B) Sensitivity (0.964) and specificity (0.709) of targeted panel-based CNAs compared to low-pass whole genome sequencing-based CNAs in the ctDNA (patients n=21)

C) The total amount of discordant segment pairs between the lpWGS and panel data (n=133), detected in 19 out of 21 patients. Discordance types are marked with different colours.

D) Correlation plot of two copy number segmentation algorithms for ctDNA landscapes. Copy number raw values (log2) from Dragen (x-axis) and ichorCNA (y-axis). Spearman's correlation.

E) Mean absolute deviations of copy number segmentation in cfDNA and FFPE samples. Dashed line represents threshold of 0.3, below which is the desired quality of the algorithm[6]. Paired Wilcoxon rank sum test.

F) Spearman's correlation of mean VAF (x-axis) and number of CNAs (y-axis, log2) in the ctDNA.

G) Waterfall plot depicting the mean VAF in the ctDNA (y-axis) of patients with and without detected CNAs (x-axis). Bars are colored by categorical mean VAF groups. Bars with asterisks (n=26) depict patients with the lpWGS data.

H) ctDNA burden (log10 hGE/ml, y-axis) between patients with mean VAF ≥ 0.015 and <0.015 (x-axis). Wilcoxon rank sum test.

I) Survival estimates for PFS in patients with a mean VAF < 0.015 (n=21).

J) Survival estimates for OS in patients with a mean VAF < 0.015 (n=21).

K) Mean change in the frequency of matching called CNAs (y-axis) in in-silico down-sampling (x-axis). The frequency of called CNA in each sample (n=11) is compared against the original sequencing file. Down-sampling dilutions are marked below L) figure.

L) The frequency of matching CNAs (y-axis) compared against the original sequencing file. In-silico dilutions are depicted in x-axis and samples (n=11) are marked with different colors.

Abbreviations: IHC: immunohistochemistry, lpWGS: low-pass whole genome sequencing, FISH: fluorescent in-situ hybridization, cfDNA: cell-free DNA, ctDNA: circulating tumor DNA, AUC: area under curve, MAD: mean absolute deviation, FFPE: formalin-fixed paraffin-embedded, VAF: variant allele frequency, CNA: copy number aberration, OS: overall survival, PFS: progression-free survival, mo: months

Figure S2. Co-occurrence of CNAs and variants

A-B) Co-occurrence of gains (A) and losses (B) with coding single nucleotide variants and small indels. The number of affected patients is in y-axis and marked on top of each bar and distinct combinations of co-occurrences are in x-axis. Different genes are marked with different colors.

Abbreviations: SNV: single nucleotide variant, del: deletion, ins: insertion

Figure S3. Minimally invasive subtype classifications

A) Sankey diagram of diagnostic subtypes and LymphGen clustering results.

B) *BCL2* and *BCL6* translocations detected by FISH (top panel) and ctDNA (lower panel). Patients with discordant FISH and ctDNA results are encircled, and the numbers are depicted at the top of the plots. Connected with dashed line a representative snapshot from IGV at chr18:63126192 from a patient with a negative *BCL2* fluorescent in-situ hybridization result, but a positive ctDNA result for a translocation call.

C) Sankey diagram of DLBclass and Lymphgen clustering results.

D) Prediction accuracies of DLBclass (upper panel) and LymphGen (lower panel) clustering in patients with tumor fraction ≥20% and <20%. Cutoffs defined according to Chapuy et al[12] study.

Abbreviations: GCB DLBCL: germinal center diffuse large B-cell lymphoma, NOS: not otherwise specified, THRLBCL: T-cell/histiocyte rich B-cell lymphoma, HGBL: high-grade B-cell lymphoma, FLG3b: follicular lymphoma grade 3B, ctDNA: circulating tumor DNA, FISH: fluorescent in-situ hybridization, CNA: copy number aberration, ns: not significant

Figure S4. Survival assessment and tumor fraction of subtype clusters

A) PFS estimate of the study cohort and validation cohort stratified by DLBclass clusters.

B) PFS estimate of the study cohort and validation cohort stratified by LymphGen clusters.

C) Tumor fraction in the study cohort and validation cohort according to the DLBclass clusters.

D) Tumor fraction in the study cohort and validation cohort according to LymphGen clusters.

E) Multivariable analyses for PFS of the clusters C2 (upper panel) and A53 (lower panel) with ctDNA concentration (running, log hGE/ml).

F) OS estimate of DLBclass subtypes assessed without CNA data in the ctDNA. Both the study and validation cohorts are included.

G) PFS estimate of DLBclass subtypes assessed without CNA data in the ctDNA. Both the study and validation cohorts are included.

Abbreviations: OS: overall survival, PFS, progression-free survival, mo: months, ctDNA: circulating tumor DNA

Figure S5. Copy number aberrations reveal clinical heterogeneity

A) Pairwise Wilcoxon rank sum comparison of tumor fractions estimated from panel-based and low-pass WGS data (n=26).

B) Waterfall plot of the validation cohort's tumor fraction. Bars in the X-axis represent individual patients and are colored by overall survival. Tumor fraction in the Y-axis. The grey dashed line corresponds to a tumor fraction of 0.03, the assay's detection limit. Mean ctDNA VAF, age, and aaIPI are annotated at the bottom of the plot for each patient, and their correlation with tumor fraction is marked on the right.

C) Survival analysis for OS for validation cohort (NLG-05). Patients are stratified by tumor fraction using a cutoff similar to that in the discovery cohort (24%).

D) Univariable analysis of tumor factor (running) for OS in the validation cohort (n=86).

E-F) Univariate Cox regression analysis for PFS of recurrent ($n \geq 5$) gains (E) and losses (F). Hazard ratio is depicted on the x-axis and p-value on the y-axis. Aberrations in genes that reached statistical significance for PFS are colored red (E) and blue (F).

G) Frequency of having one or more high-risk CNAs between clinical risk factors (X-axis). Number of patients as Y-axis. Bars are colored according to the number of patients harboring one or more prognostic CNAs. aaIPI: Kruskal-Wallis test, age and B-symptoms: Chi-square test, LDH, Stage: t-test.

Abbreviations: ns: not significant, lpWGS: low-pass whole genome sequencing, PFS: progression-free survival, mo: months, OS: overall survival, aaIPI: age-adjusted International Prognostic Index, ctDNA: circulating tumor DNA, VAF: variant allele frequency, CNA: copy number aberration, HR: hazard ratio, CI: confidence interval, LDH: lactate dehydrogenase

Figure S6. Survival estimate of cumulative high-risk CNAs

A) Survival estimates for OS of patients stratified by the cumulative number of prognostic CNAs

B) Survival estimates for PFS of patients stratified by the cumulative number of prognostic CNAs

C) Number of high-risk CNAs in DLBclass molecular subtypes.

D-E) Multivariable analysis for OS (D) and PFS (E): age, aaIPI, ctDNA concentration (running), and the presence of any high-risk CNA (n=40).

Abbreviations: OS: overall survival, PFS: progression-free survival, mo: months, ctDNA: circulating tumor DNA, CNA: copy number aberration, aaIPI: age-adjusted International Prognostic Index, HR: hazard ratio

Figure S7. *TP53* loss in the ctDNA

A) FISH-informed *TP53*/17p status compared to segmentation values from publicly available CNA segmentation pipeline (ichorCNA). Blue and yellow dots represent losses and neutral regions in ichorCNA data, respectively. Similar cutoffs for calling losses are used as in the main data (Dragen pipeline).

B) Comparison of copy number segment means (Y-axis) between the Dragen and ichorCNA pipelines at the *TP53* region. Points represent patients and are colored by *TP53* status.

C) Coding *TP53* variants in the ctDNA of patients with VAF ≥ 0.015. Variant data points are colored by their pathogenicity. *TP53* copy number loss, assessed by FISH or ctDNA, is depicted by a circle.

D) Comparison of *TP53* copy number segment means (log2) in ctDNA between P53 IHC staining statuses from lymphoma tissues.

E) ctDNA concentration in patients with FISH-positive *TP53*/17p result. Patients with detected and undetected *TP53* loss in ctDNA are colored blue and yellow, respectively. Wilcoxon rank sum test comparing the ctDNA concentration between the ctDNA *TP53* groups.

F) Tumor fraction in the patients with FISH-positive *TP53*/17p result. Patients with detected and undetected *TP53* loss in the ctDNA are colored blue and yellow, respectively. Wilcoxon rank sum test comparing tumor fraction between the ctDNA *TP53* groups.

G) Survival analysis of PFS in patients stratified by ctDNA *TP53* CNA status (n=102).

H) Survival analysis of PFS in patients stratified by FISH-informed p53/17p status (n=114).

I) Multivariable analyses for OS of age (running), aaIPI (running), and FISH-informed *TP53*/17p status (categorical; positive n=19) (top panel), age, aaIPI, ctDNA concentration (running), and ctDNA *TP53* loss (n=11) (middle panel), age, aaIPI, tumor fraction (running), and ctDNA *TP53* loss (n=11) (lower panel)

J) Survival analysis for OS of validation cohort (LBC-05) stratified by ctDNA *TP53* copy number status.

K) Survival analysis for OS of the patients stratified by co-occurrence of non-silent *TP53* variant and TP53 loss.

Abbreviations: FISH: fluorescent in-situ hybridization, CNA: copy number aberration, N/A: not available, IHC: immunohistochemistry, PFS: progression-free survival, mo: months, ctDNA: circulating tumor DNA, VAF: variant allele frequency, HR: hazard ratio, aaIPI: age-adjusted International Prognostic Index, OS: overall survival

Figure S8. Population dynamics of R/R lymphoma clones

A) Mean cancer cell fractions (Y-axis) of distinct clones (X-axis) in pretreatment samples. Patients are marked with different colors.

B) Mean cancer cell fractions (Y-axis) of distinct clones (X-axis) at R/R samples. Patients are marked with different colors.

C) Paired cancer cell fractions of clones from pretreatment and R/R samples. Wilcoxon rank sum test significance levels of pair cancer cell fractions are depicted on top. Distinct clones are marked with different colors.

Abbreviations: CCF: cancer cell fraction, ns: not significant, R/R: relapsed/refractory

Supplementary Figure 1.

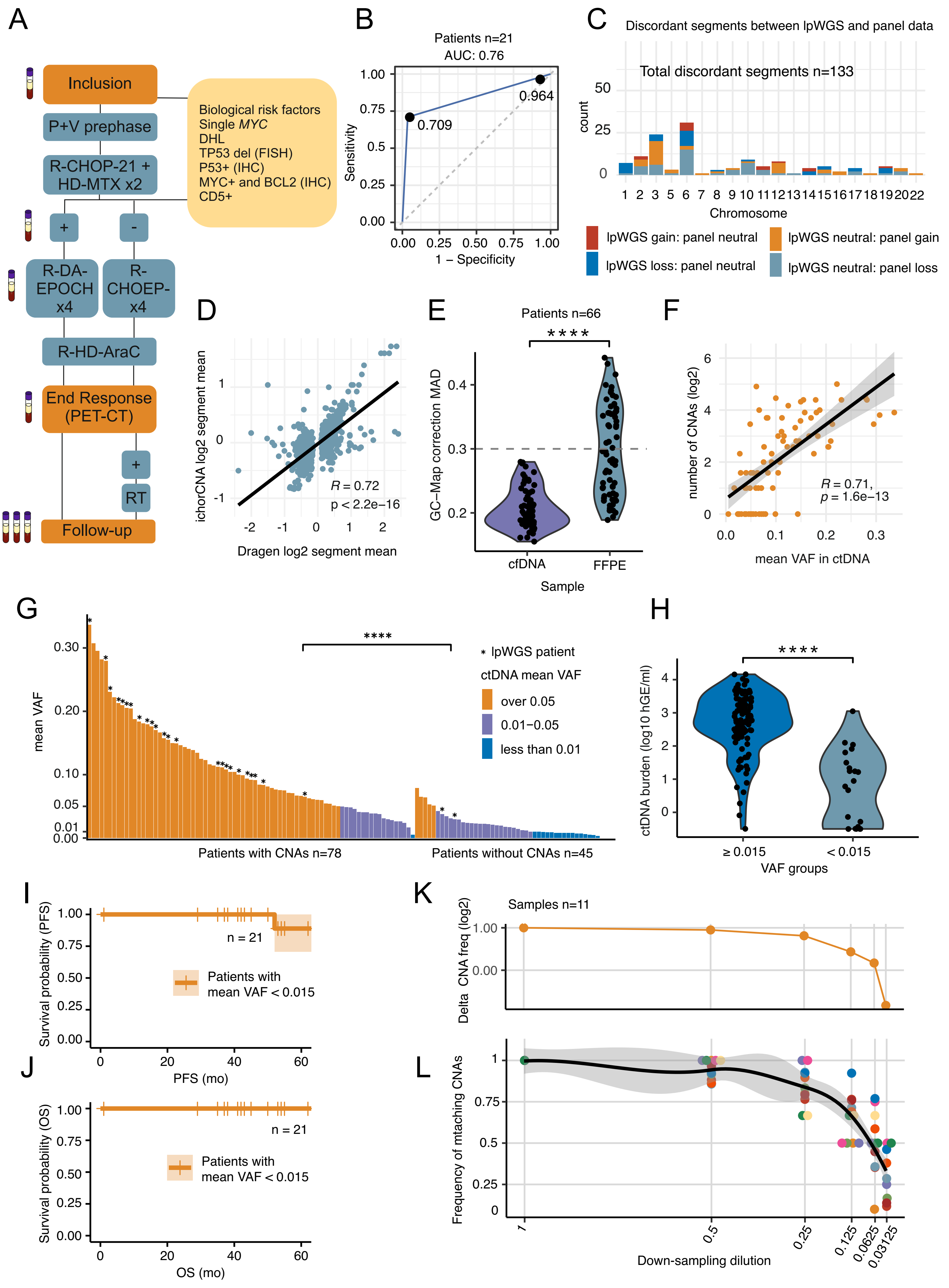

Supplementary Figure 2.

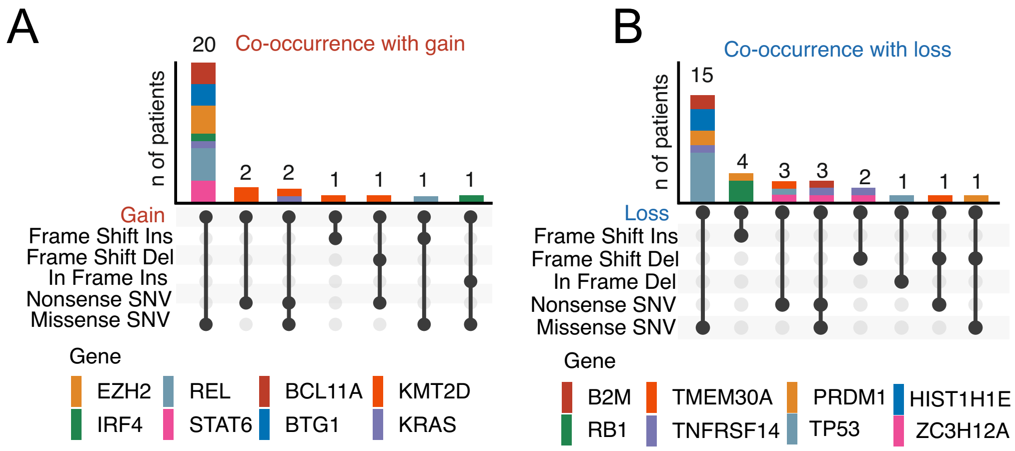

Supplementary Figure 3.

Arffman et al.

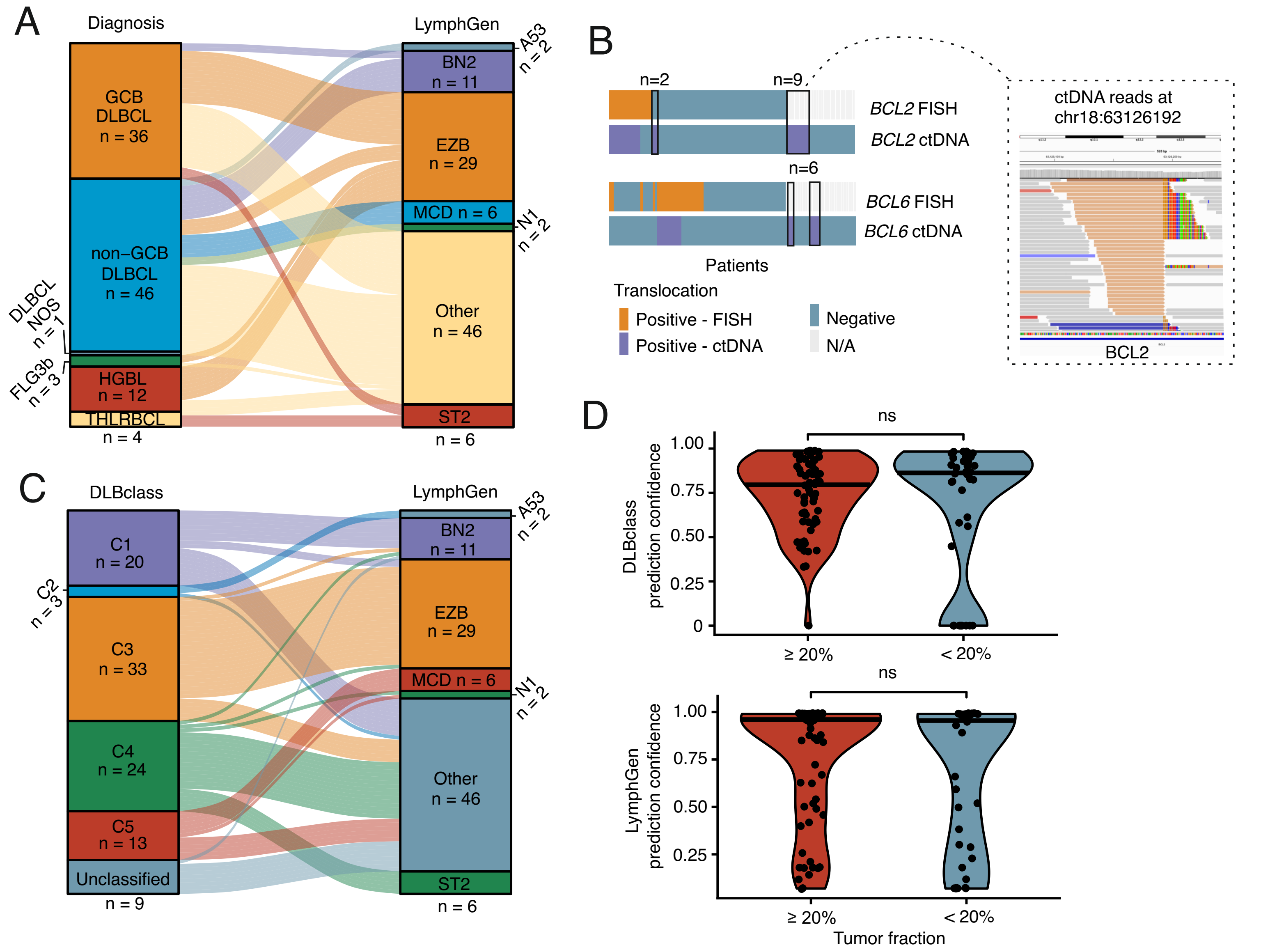

Supplementary Figure 4.

Arffman et al.

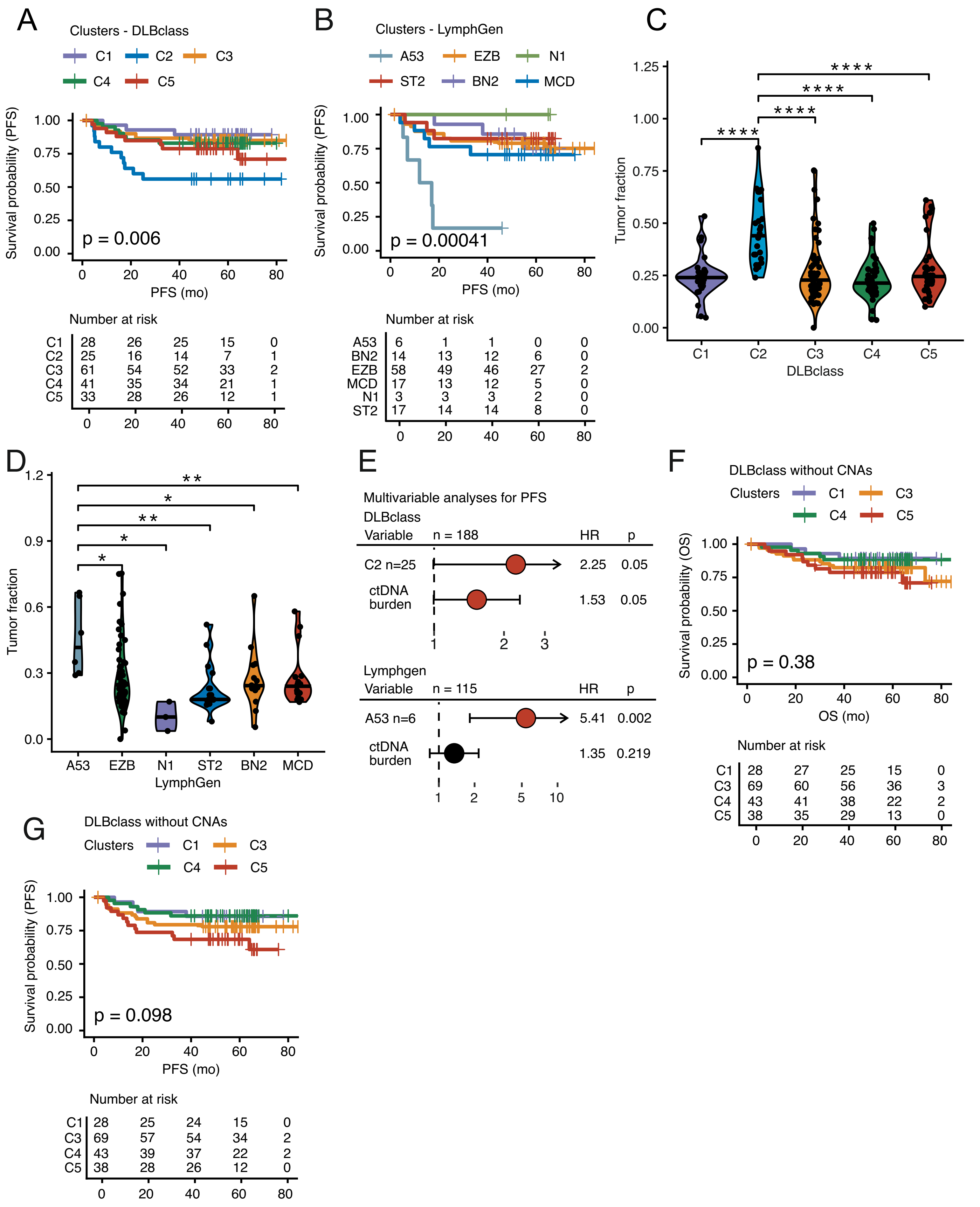

Supplementary Figure 5.

Arffman et al.

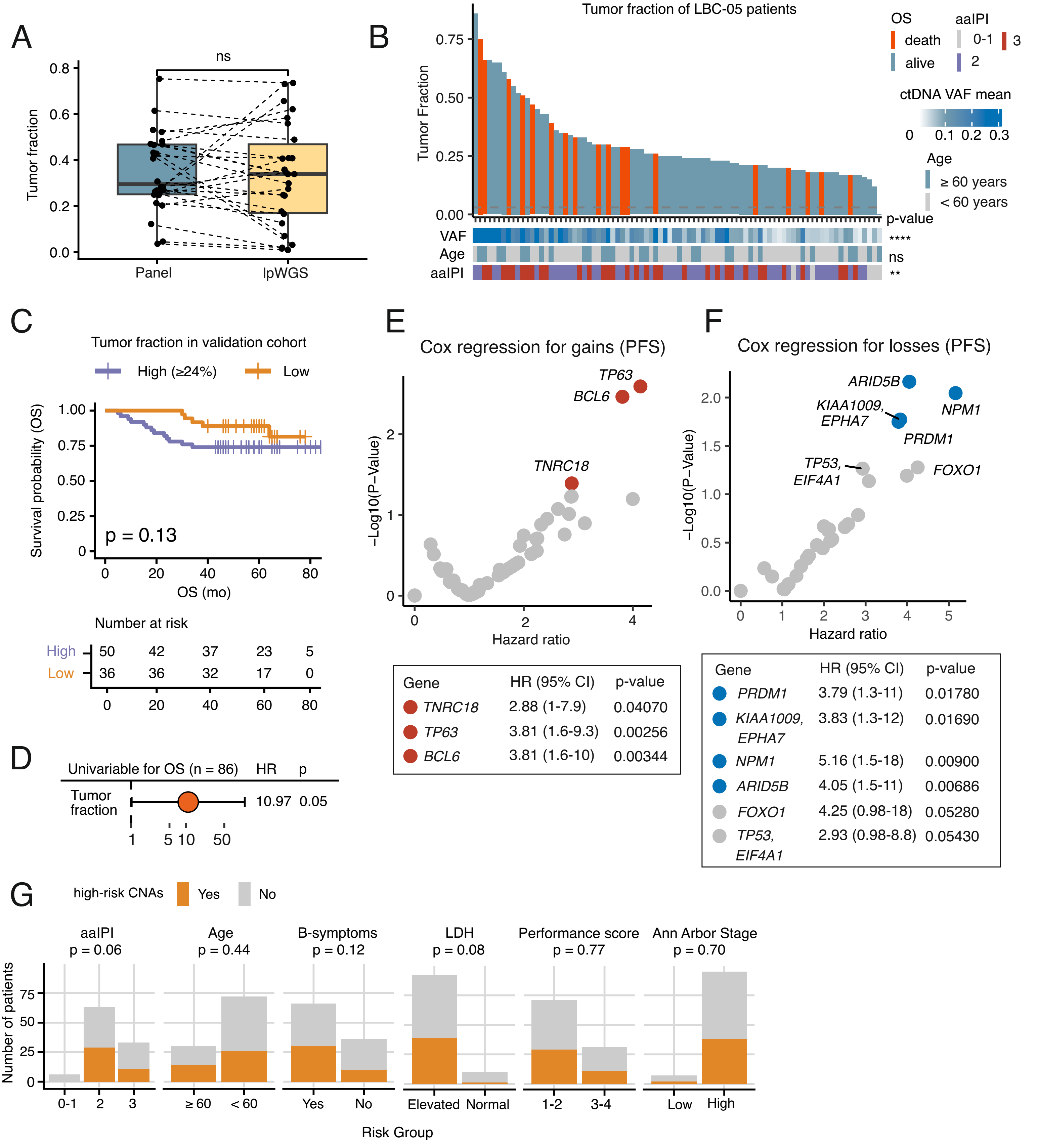

Supplementary Figure 6.

Arffman et al.

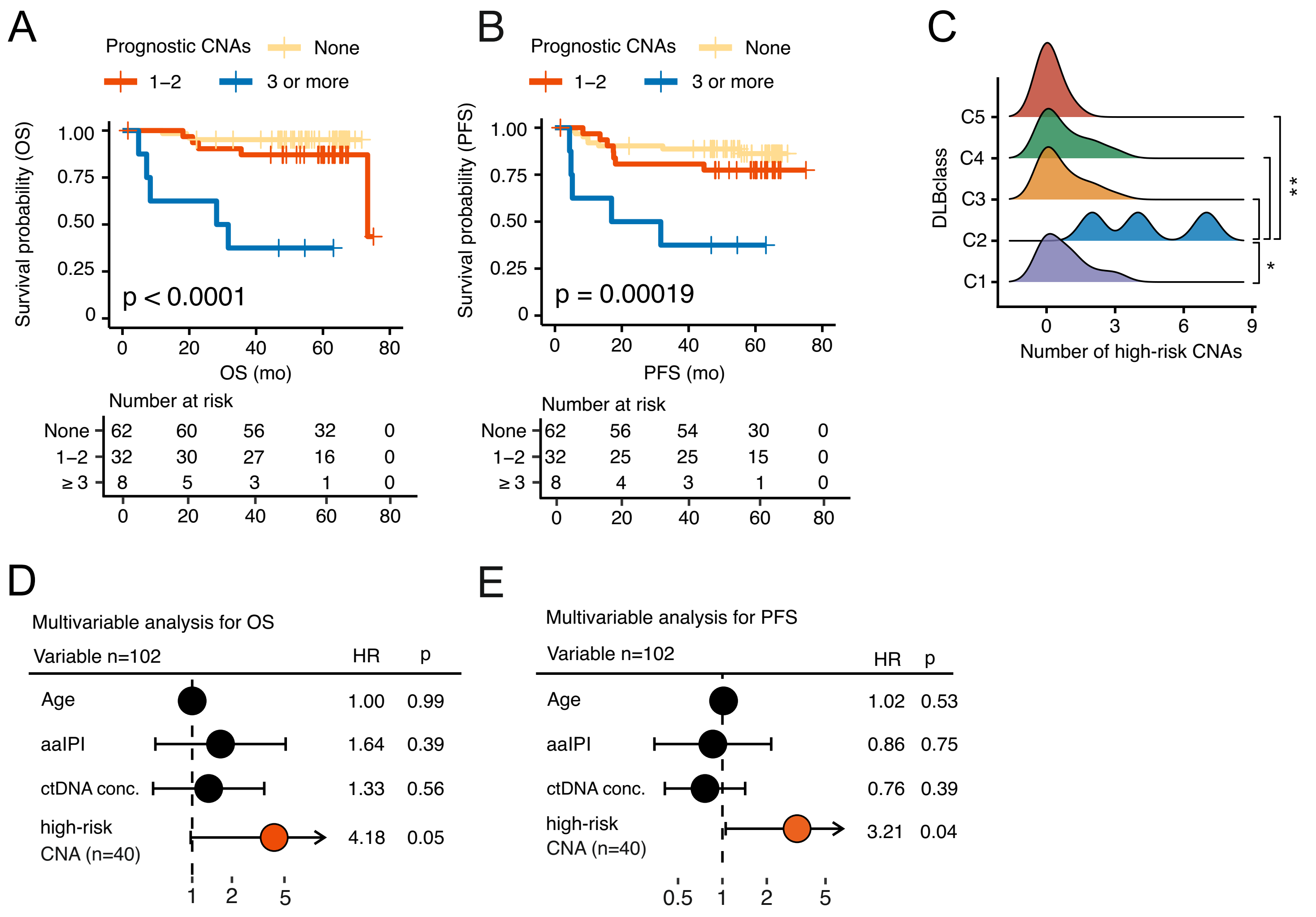

Supplementary Figure 7. Arffman et al.

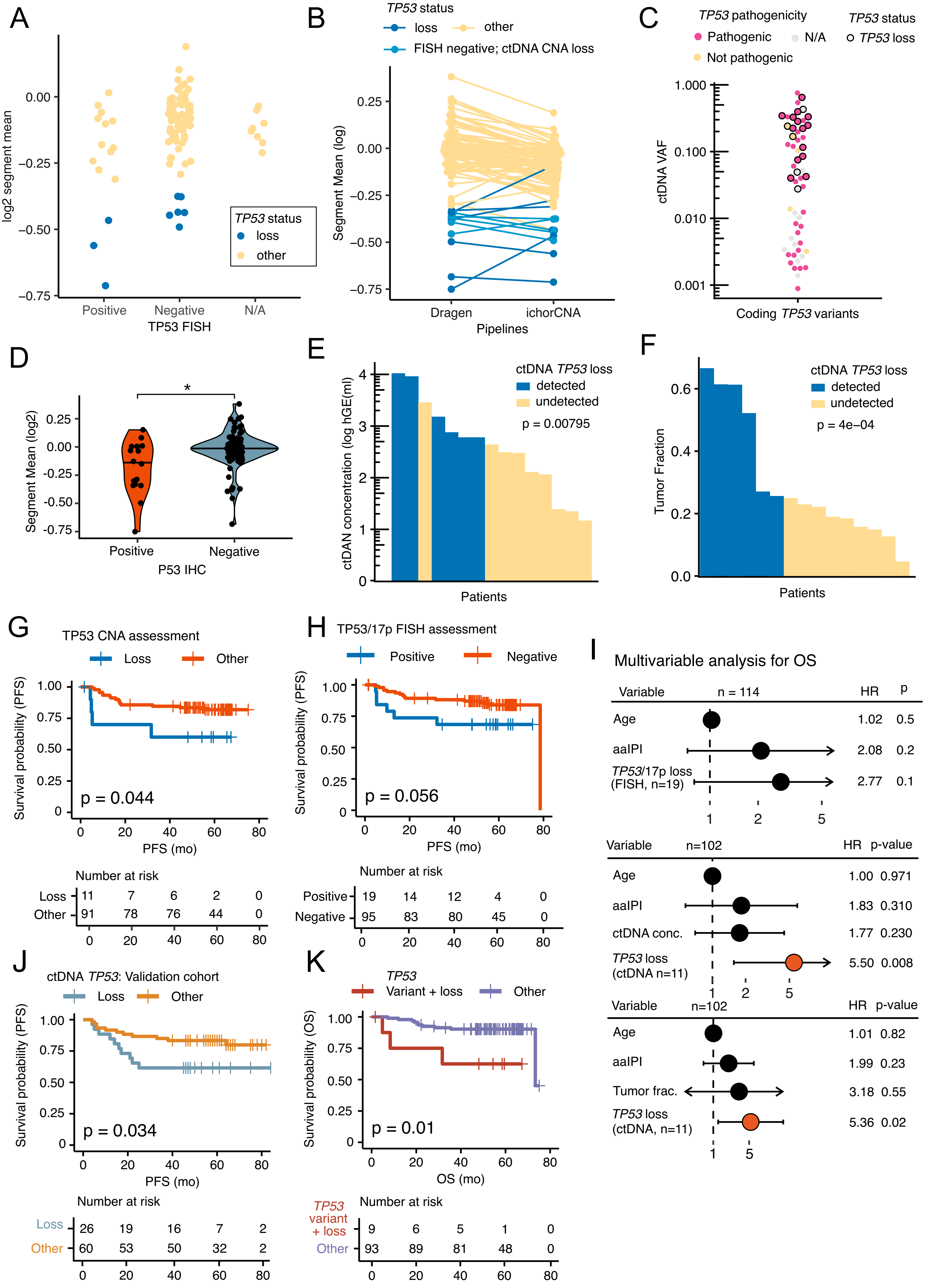

Supplementary Figure 8.

Arffman et al.

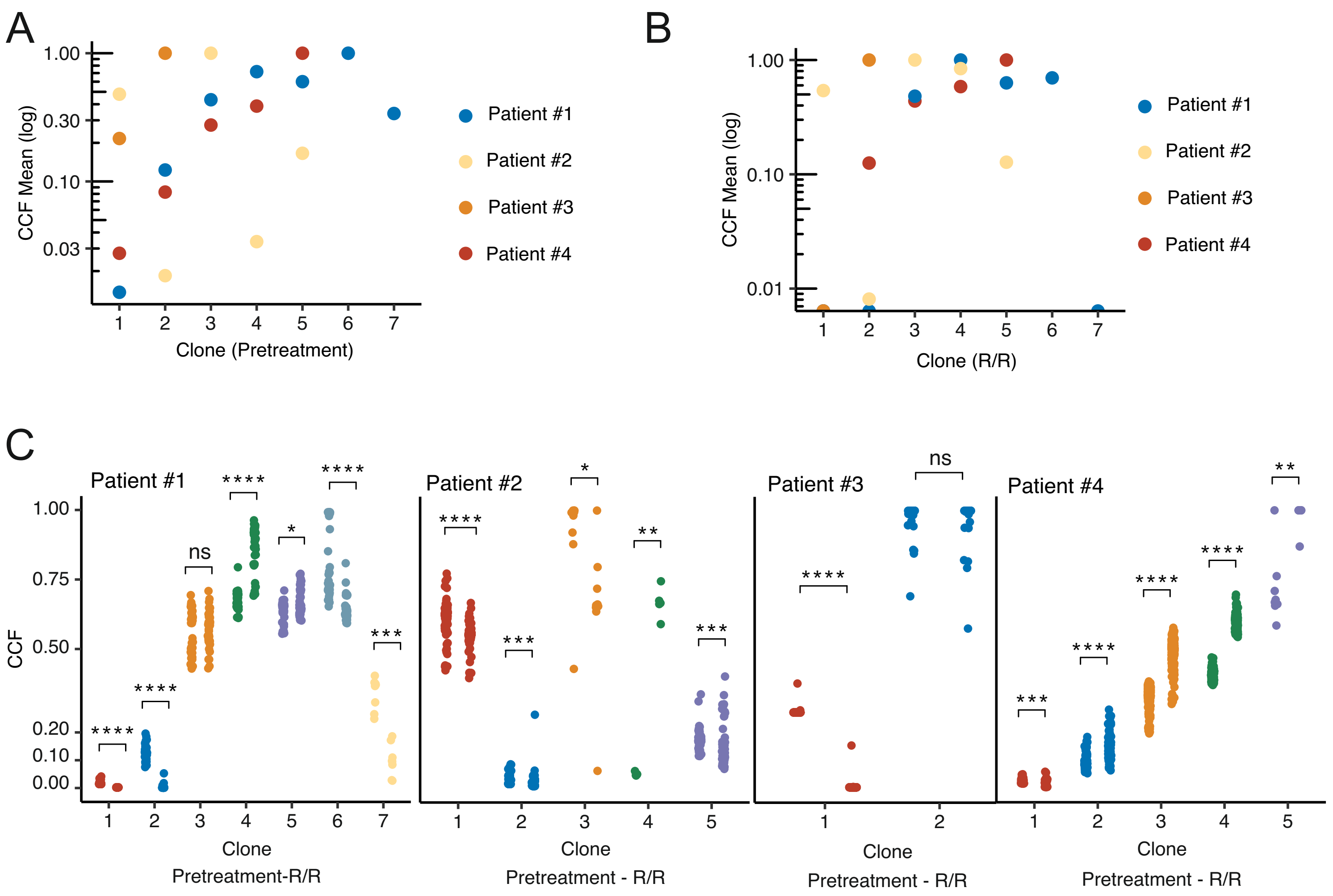

Supplement: Supplementary file 1 — Supplementary material [file 41375_2026_2955_MOESM1_ESM.pdf]
